# Twenty‐four‐month outcomes from a cluster‐randomized controlled trial of extending antiretroviral therapy refills in ART adherence clubs

**DOI:** 10.1002/jia2.25649

**Published:** 2020-12-19

**Authors:** Tali Cassidy, Anna Grimsrud, Claire Keene, Keitumetse Lebelo, Helen Hayes, Catherine Orrell, Nompumelelo Zokufa, Tabitha Mutseyekwa, Jacqueline Voget, Rodd Gerstenhaber, Lynne Wilkinson

**Affiliations:** ^1^ Médecins Sans Frontières Khayelitsha South Africa; ^2^ Department of Public Health Medicine School of Public Health and Family Medicine University of Cape Town Cape Town South Africa; ^3^ International AIDS Society Cape Town South Africa; ^4^ Western Cape Government Department of Health Cape Town South Africa; ^5^ Department of Medicine Faculty of Health Sciences Cape Town South Africa; ^6^ The Desmond Tutu HIV Centre Institute for Infectious Disease and Molecular Medicine University of Cape Town Cape Town South Africa; ^7^ Center for Infectious Disease and Epidemiological Research School of Public Health and Family Medicine University of Cape Town Cape Town South Africa

**Keywords:** multi‐month dispensing, differentiated service delivery, HIV, adherence clubs, patient‐centred, ART retention in care

## Abstract

**Introduction:**

The antiretroviral therapy (ART) adherence club (AC) model has supported clinically stable HIV patients’ retention with group ART refills and psychosocial support. Reducing visit frequency by increasing ART refills to six months could further benefit patients and unburden health systems. We conducted a pragmatic non‐inferiority cluster randomized trial comparing standard of care (SoC) ACs and six‐month refill intervention ACs in a primary care facility in Khayelitsha, South Africa.

**Methods:**

Existing community‐based and facility‐based ACs were randomized to either SoC or intervention ACs. SoC ACs met five times annually, receiving two‐month refills with a four‐month refill over year‐end. Blood was drawn at one AC visit with a clinical assessment at the next. Intervention ACs met twice annually receiving six‐month refills, with an individual blood collection visit before the annual clinical assessment AC visit. The first study visits were in October and November 2017 and participants followed for 27 months. We report retention in care, viral load completion and viral suppression (<400 copies/mL) 24 months after enrolment and calculated intention‐to‐treat risk differences for the primary outcomes using generalized estimating equations specifying for clustering by AC.

**Results:**

Of 2150 participants included in the trial, 977 were assigned to the intervention arm (40 ACs) and 1173 to the SoC (48 ACs). Patient characteristics at enrolment were similar across groups. Retention in care at 24 months was similarly high in both arms: 93.6% (1098/1173) in SoC and 92.6% (905/977) in the intervention arm, with a risk difference of −1.0% (95% CI: −3.2 to 1.3). The intervention arm had higher viral load completion (90.8% (999/1173) versus 85.1% (887/977)) and suppression (87.3% (969 /1173) versus 82.6% (853/977)) at 24 months, with a risk difference for completion of 5.5% (95% CI: 1.5 to 9.5) and suppression of 4.6% (95% CI: 0.2 to 9.0).

**Conclusions:**

Intervention AC patients receiving six‐month ART refills showed non‐inferior retention in care, viral load completion and viral load suppression to those in SoC ACs, adding to a growing literature showing good outcomes with extended ART dispensing intervals.

## 1 Introduction

South Africa has the largest antiretroviral therapy (ART) programme in the world, with more than five million people on ART at the end of 2019. With approximately 7.6 million people living with HIV in the country, this represents 65.8% ART coverage [1, 2]. In order to retain those already on treatment and attract those that are not, ART service delivery needs to adapt to be more client‐centred. Differentiated service delivery (DSD) has emerged as an approach for HIV programmes seeking to better serve the needs of people living with HIV, reduce unnecessary burdens on the health system, and improve client outcomes [3].

DSD for HIV treatment focuses on the ART delivery component of the HIV care cascade adapting service delivery for clinically stable patients including through less frequent facility visits enabled by longer drug refills. In sub‐Saharan Africa, countries have implemented DSD models for HIV treatment including individual models, both facility‐based and out‐of‐facility, as well as group models that are either client‐led or managed by healthcare workers [4, 5]. ART adherence clubs (AC) are a healthcare worker‐managed group model that has been adopted as national policy in many countries, including South Africa. South Africa’s 2020 national AC standard operating procedure (SOP) describes an AC as a group of 10 to 30 clinically stable ART patients, facilitated by a lay healthcare worker, that meet at their facility or in their community every two to three months for a group support session, brief symptom check and distribution of pre‐packed ART [6]. Patients generally see a clinician annually for a comprehensive clinical consultation, but are referred for additional clinical care if necessary. ACs have shown good retention and viral suppression outcomes both at site level and at scale and are acceptable to patients [7, 8, 9, 10, 11, 12, 13, 14].

There is a need to continually investigate the adaptations of differentiated ART delivery models to further increase convenience and access for patients as well as efficiency for healthcare systems. Reducing the frequency of AC visits by increasing the amount of ART dispensed at each refill could convenience further. Such an adaption could reduce costs for patients and increase nurse capacity for those still needing to start ART or requiring more intensive clinical care [15, 16].

The World Health Organization’s (WHO) 2016 ART guidelines already recommend 3‐ to 6‐month ART refills for clinically stable patients [17]. This is supported by the U.S. President's Emergency Plan for AIDS Relief (PEPFAR) COP 2020 guidance [18]. Prior to 2020, Ethiopia [19], Guinea [20], Malawi [21], Namibia [22] and Zambia [23] had endorsed and were implementing six‐month ART refills for stable adults. In 2020, in response to the COVID‐19 pandemic, Democratic Republic of Congo [24], Eswatini [25], Liberia [26], South Sudan [27], Tanzania [28] and Zimbabwe [29] also prioritized six‐month ART refills in their interim guidance to reduce the frequency of visits to health facilities. It remains uncertain whether these emergency measures will be adopted into DSD country policy and/or implementation plans in a post‐COVID era. Recent trials in Lesotho, Malawi, Zambia and Zimbabwe evaluated six‐month ART refills in community and individual models [30, 31, 32].

We conducted a study of six‐month ART refills within the widely endorsed and implemented AC DSD model. We describe 24‐month results from this cluster‐randomized non‐inferiority trial investigating the hypothesis that extended ART dispensing intervals among existing AC patients will result in non‐inferior retention in care and viral load (VL) outcomes.

## 2 Methods

### 2.1 Study population and setting

The study took place at the Western Cape Provincial Department of Health (WCDoH)‐run Ubuntu ART clinic at the Site B Community Health Center in Khayelitsha, a peri‐urban area in Cape Town, South Africa, home to approximately 500 000 people [33, 34]. The Ubuntu clinic started ACs in 2007 [35]. ACs are managed by WCDoH staff with AC facilitators employed by a PEPFAR‐supported non‐governmental organization. At the start of study recruitment, 44.2% of the clinic’s 10 252 ART patients received their ART in ACs.

### 2.2 Study design, eligibility, enrolment and randomization

This was a pragmatic, unblinded, cluster‐randomized, non‐inferiority trial comparing six‐month to two‐month ART dispensing intervals in ACs over two years.

Participants eligible for AC enrolment were >18 years; not pregnant, not experiencing an opportunistic infection, on ART for more than six months with one VL below 400 copies/ml, able to provide consent, and already in an existing AC (other than family, youth and evening ACs).

Recruitment for the trial began in February 2017 and the first study visits were AC visits in October‐November 2017. When ≥90% of AC members present voted to participate in the study, the AC was eligible for inclusion. Members were individually consented and offered to transfer to another club if they did not wish to participate. Eligible and consenting ACs were randomized 45:55 to the intervention or standard of care (SoC) using the *Randomize* package in Stata, ensuring balance between community and facility ACs in each arm, by a team member not involved in recruitment or enrolment. The number of ACs assigned to the intervention arm was limited by ART drug costs funded by the study. Study staff drew an envelope containing the randomization outcome at the post‐consent AC meeting and if once again ≥90% of members voted for continued study participation, the AC was enrolled in the study. Further detail can be found in the published protocol [36].

### 2.3 Intervention description

The SoC (two‐monthly refills) and the intervention (six‐monthly refills) ACs are described in Table 1.

**Table 1:** Comparison of SoC ACs and intervention ACs

	Standard of care ACs	Intervention ACs
Frequency of AC visits	2 monthly × 4+ 4 monthly × 1 (5 per year)	6 monthly (2 per year)
ART dispensing interval	2 monthly × 4 4 monthly × 1 (5 per year)	6 monthly (2 per year)
Frequency of clinical consultations	12‐monthly	12‐monthly
Frequency of routine bloods	12‐monthly	12‐monthly
Timing of routine lab tests	Part of AC visit	An additional individual visit, scheduled two to six weeks before the AC visit
Treatment “buddies”	Allowed to collect at every other visit	Not permitted
ART packing and dispensing	Pre‐packed by central dispensing unit, delivered to the *clinic pharmacy*, dispensed at AC visit	Pre‐packed at the clinic pharmacy with support from *research staff*, dispensed at AC visit
Standard number of contacts per year	5	3 (2 within the AC and 1 individual for routine bloods)
Size of ACs	Groups of 25 to 30	Groups of 25 to 30
Peer‐based support	Strong emphasis	Strong emphasis
Patient self‐management	Strong emphasis	Strong emphasis
Management of clinical complications	Up‐referral to clinic clinicians – patient exits AC and returns to routine clinic appointments	Up‐referral to clinic clinicians – patient exits AC and returns to routine clinic appointments
Grace period	5 days	5 days
Minimum number of contacts for the patient per year	3 (could send a “treatment buddy” to collect ART twice)	3

#### 2.3.1 Standard of care ACs

The WCDoH ACs, previously described [37, 38], met five times per annum. Four AC meetings dispensed two months of ART and one meeting before year‐end dispensed four months of ART to support travel over the holiday period. In the first year of the study (2018), all WCDoH ACs including the SOC ACs received an additional once‐off four‐month ART refill due to the water crisis. VL was taken at one AC visit annually with the next AC visit at the clinic including an individual clinician consultation.

#### 2.3.2 Intervention ACs

Intervention ACs met twice a year and were dispensed six months of ART. One of these visits included a clinical consultation. Intervention patients were given a VL appointment date and allowed to come anytime two to six weeks before their clinical consultation AC visit. Those who did not come for their VL appointment had their VL taken at their clinical consultation AC visit and those with high VLs were recalled. Intervention ACs facilitators remained the same as before the study. VLs and clinical consultations were undertaken by a study nurse.

**Figure 1:**
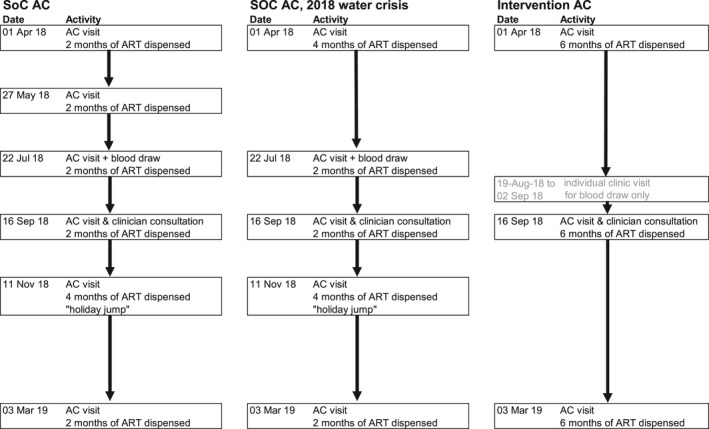
Comparative SOC and Intervention AC schedule examples.

Figure 1 illustrates a comparative example of SOC and Intervention AC’s annual schedule.

### 2.4 Data collection

Retention data were collected from routine AC registers completed by facilitators and VL data from laboratory data. A study data capturer captured visit and laboratory data into REDCap [36, 39].

### 2.5 Study outcomes

The primary outcomes were 24‐month retention in care, VL completion and suppression. Secondary outcomes were 24‐month retention in ACs, and 12‐month retention in care and VL completion and suppression.

We also reported reasons for referral from AC to clinic care and the timing of blood draws in relation to clinical consultations to investigate operational concerns regarding the availability of VL results at the annual clinical consultation.

### 2.6 Outcome definitions

“Retention in care” at 24 months was defined as any ART collection (AC or clinic visit) at 24 months or within three months thereafter. If the last documented AC or clinic attendance was 12 months or more after the participant’s first study visit, they were considered retained in care at 12 months.

“Retention in AC care” was defined as attending the scheduled AC 24‐month visit. AC patients who missed an AC visit by more than five days but were allowed to return to the AC were not considered retained.

“VL completion” was defined as having a VL result within 12 months of the first study visit (12‐month VL completion) or between 12 and 24 months of the first study visit (24‐month VL completion).

“VL suppression” was defined as a VL below 400 copies/mL.

### 2.7 Analysis

Retention and VL outcomes were calculated as a proportion of total participants enrolled (intention‐to‐treat, ITT). VL completion and suppression were also presented as a proportion of those enrolled, excluding those transferring to another clinic (modified intention‐to‐treat, mITT), and VL suppression as a proportion of those with VL completed.

Risk differences were calculated using binomial generalized estimating equations, using robust standard errors and specifying clustering by AC. Outcomes were not adjusted for baseline characteristics, but sensitivity analyses were conducted for patients initiating ART in the previous three years, and stratifying by documented VL suppression at baseline and facility versus community ACs. All analysis was conducted using Stata 15 [40].

### 2.8 Sample size

The pre‐specified non‐inferiority limit was 5% [36]. ITT sample size calculations were updated to reflect actual enrolment, intra‐cluster correlations, and observed 24‐month outcome proportions in the SoC arm. The study was powered at 80% to detect a 3.1% or more reduction in retention in care, a 6.5% reduction in VL completion, and a 6.7% reduction in VL suppression in the intervention AC patients (Table S1).

### 2.9 Ethics

Ethics approval was granted by the University of Cape Town (HREC 652/2016), Médecins Sans Frontières Ethics Review Board (protocol #1639) and WCDoH (WC_2016RP41_532). The trial was registered retrospectively with the Pan African Clinical Trial Registry (PACTR201810631281009) on 10 October 2018.

## 3 Results

### 3.1 Enrolment

**Figure 2:**
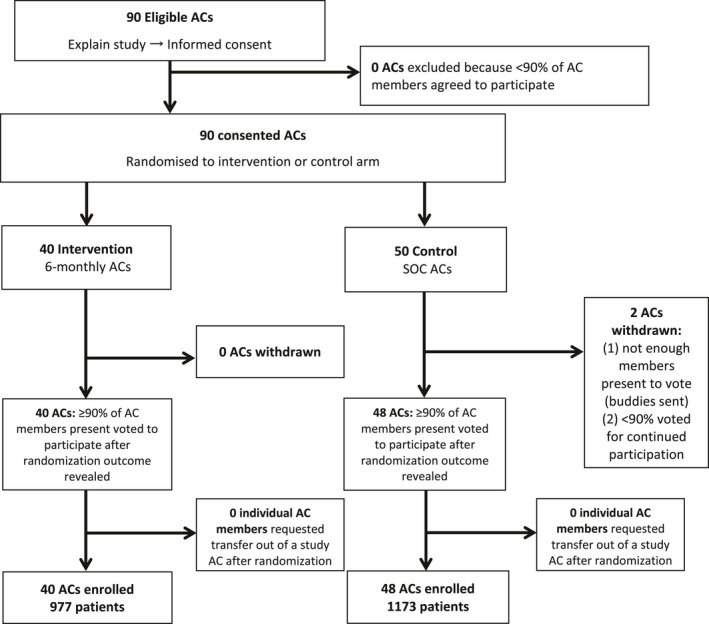
Study recruitment and enrolment flowchart.

All 90 sampled ACs were eligible for study inclusion. After randomization, two ACs withdrew from the SoC: one did not have enough members present to vote for continued participation (most had sent buddies to collect treatment), and in the other <90% of members present voted for continued participation. No individual AC members asked to be transferred out of a participating AC. In total, 40 ACs were assigned to the intervention (n = 977 patients) and 48 ACs were assigned to the SoC (n = 1173) (Figure 2).

### 3.2 Patient characteristics

A total of 2150 patients were enrolled in the trial and characteristics were similar in the two arms. There were fewer men than women in both arms (23.7% in SoC and 21.8% in intervention). Participants were on ART for a median of 7.3 years (interquartile range (IQR): 4.7 to 10.2 years) at study start and had a median age of 41.4 years (IQR 36.5 to 47.2 years). The majority (86.9%) were on a fixed‐dose regimen and had a suppressed VL (95.1%) in the 18 months prior to enrolment (Table 2).

**Table 2:** Patient characteristics at study enrolment

	SoC AC patients (n = 1173)	Intervention AC patients (n = 977)	Total (n = 2150)
Number of ACs	48	40	98
Community ACs (vs. facility based ACs), n (%)	567 (48.3%)	434 (44.4%)	1001 (46.6%)
Male, n (%)	278 (23.7%)	213 (21.8%)	491 (22.8%)
Time on ART			
Median time on ART, years (IQR)	7.1 (4.5 to 10.5)	7.4 (5.0 to 10.0)	7.3 (4.7 to 10.2)
On ART <3 years, n (%)	120 (10.2%)	93 (9.5%)	213 (9.9%)
Era of ART initiation (by CD4 count eligibility criteria), n (%)			
<200 (before August 2011)	672 (57.3%)	611 (62.5%)	1283 (59.7%)
<350 (Aug 2011 to 31 Dec 2014)	398 (33.9%)	280 (28.7%)	678 (31.5%)
<500 (1 Jan 2015 to 31 Aug 2016)	99 (8.4%)	84 (8.6%)	183 (8.5%)
All eligible (After 1 Sept 2016)	4 (0.3%)	1 (0.1%)	5 (0.2%)
Age, n(%)			
18 to 34 years	221 (18.8%)	172 (17.6%)	393 (18.3%)
35 to 49 years	759 (64.7%)	646 (66.1%)	1405 (65.3%)
50 + years	193 (16.5%)	159 (16.3%)	352 (16.4%)
Median age, years (IQR)	41.4 (36.2 to 47.1)	41.5 (36.6 to 47.3)	41.4 (36.5 to 47.2)
On fixed dose regimen^a^, n (%) (TDF/FTC/EFV)	1027 (87.6%)	841 (86.1%)	1868 (86.9%)
Baseline VL (0 to 18 months before first study visit), n (%)			
Suppressed^b^	1120 (95.5%)	925 (94.7%)	2045 (95.1%)
Unsuppressed	19 (1.6%)	18 (1.8%)	37 (1.7%)
Incomplete	34 (2.9%)	34 (3.5%)	68 (3.2%)

AC, adherence club; ART, antiretroviral therapy; IQR, interquartile range; SoC, standard of care; VL, viral load.

^a^ Fixed dose regimen was tenofovir (TDF), emtricitabine (FTC) and efavirenz (EFV) (TDF/FTC/EFV)

^b^ Suppressed was defined as having a documented viral load below 400 copies/mL in the past 18 months.

### 3.3 Outcomes

Retention in care at 12 and 24 months was similar between arms with a risk difference of −1.0% (95% confidence interval (CI): −3.2 to 1.3) at 24 months. Over 24 months, 117 were lost to follow‐up, and 27 transferred out. There was no significant difference in retention, death, transfers or loss to follow‐up at 12 or 24 months. The exception was retention in AC care at 24 months where a greater proportion of participants were still in intervention ACs (75.0%) compared to SoC ACs (64.1%), with a risk difference of 10.7% (95% CI: 5.1 to 16.4%) (Table 3).

**Table 3:** Retention outcomes at 12 and 24 months

	SoC ACs n (%)	Intervention ACs n (%)	ICC	Risk difference^a^ (95% CI), Intervention versus SoC
12 months				
Retained in care	1146 (97.7%)	953 (97.5%)	<0.001	−0.2% (−1.4 to 1.1)
Deceased	0 (0.0%)	2 (0.2%)	<0.001	0.2% (−1.0 to 1.5)^b^
Transferred out	6 (0.5%)	2 (0.2%)	<0.001	−0.3% (−0.8 to 0.2)
Lost to follow‐up	21 (1.8%)	20 (2.0%)	0.005	0.3% (−1.0 to 1.5)
24 months				
Retained in care	1098 (93.6%)	905 (92.6%)	0.005	−1.0% (−3.2 to 1.3)
Deceased	3 (0.3%)	2 (0.2%)	<0.001	−0.1% (−0.4 to 0.3)
Transferred out	13 (1.1%)	14 (1.4%)	0.008	0.3% (−0.7 to 1.3)
Lost to follow‐up	59 (5%)	56 (5.7%)	0.002	0.7% (−1.3 to 2.7)
Retained in AC care	752 (64.1%)	733 (75.0%)	0.062	10.7% (5.1 to 16.4)

AC, adherence clubs; ICC, intra‐cluster correlation; SoC, standard of care.

^a^ Calculated using generalized estimating equations using robust standard errors and specifying clustering by adherence club

^b^ Calculated using two‐sample test of proportions because there were no events in the SoC arm.

The majority (65.7%) of patients not retained in AC care at 24 months were late for their visit (Table S2) and therefore up‐referred to clinician‐based care as per the WCDoH AC SOP. Out of the patients not considered retained in AC care for this analysis, 40.9% of SoC and 20.1% of intervention patients missed an AC visit and then returned to AC care, contrary to the WCDoH AC SOP.

In the ITT analysis, VL completion and suppression were higher in the intervention arm at both 12 months and 24 months. VL completion was 98% versus 94% at 12 months (risk difference 3.8%, 95% CI 0.3 to 7.2) and 90.8 versus 85.2% at 24 months (risk difference 5.5%, 95% CI 1.5 to 9.5) in the intervention compared to the control. Similarly, the risk difference in VL suppression was 5.1% (95% CI 1.3 to 8.9%) at 12 months and 4.6% (95% CI 0.2 to 9.0%) at 24 months. In the mITT analysis, the intervention arm still compared favourably to the SoC. VL suppression as a proportion of those with a VL completed did not differ between the intervention and control ACs (Table 4, Figure S1).

**Table 4:** Viral load outcomes at 12 and 24 months, with risk differences calculated using generalized estimating equations

	Intention to treat^a^				Modified intention to treat^b^				Completed viral load			
12 months												
	SoC (N = 1173)	Intervention (N = 977)	ICC	RD (95% CI)	SoC (N = 1167)	Intervention (N = 975)	ICC	RD (95% CI)	SoC (N = 1107)	Intervention (N = 956)	ICC	RD (95% CI)
	n (%)	n (%)			n (%)	n (%)			n (%)	n (%)		
Viral load completed	1107 (94.4%)	956 (97.9%)	0.166	3.8% (0.3 to 7.2)	1106 (94.8%)	955 (97.9%)	0.178	3.5% (0 to 6.9)		—		—
Viral load suppressed	1065 (90.8%)	933 (95.5%)	0.111	5.1% (1.3 to 8.9)	1064 (91.2%)	932 (95.6%)	0.115	4.8% (1 to 8.6)	1065 (96.2%)	933 (97.6%)	0.019	1.5% (−0.3 to 3.2)

24 months												
	SoC (N = 1173)	Intervention (N = 977)	ICC	RD (95% CI)	SoC (N = 1160)	Intervention (N = 963)	ICC	RD (95% CI)	SoC (N = 999)	Intervention (N = 887)	ICC	RD (95% CI)
Viral load completed	999 (85.2%)	887 (90.8%)	0.062	5.5% (1.5 to 9.5)	996 (85.9%)	879 (91.3%)	0.061	5.3% (1.4 to 9.2)		—		—
Viral load suppressed	969 (82.6%)	853 (87.3%)	0.058	4.6% (0.2 to 9.0)	967 (83.4%)	847 (88.0%)	0.059	4.5% (0.2 to 8.9)	969 (97%)	853 (96.2%)	0.008	−0.8% (−2.5 to 0.9)

CI, confidence interval; ICC, intra‐cluster correlation; RD, risk difference; SoC, standard of care.

^a^ All enrolled are included in the denominators

^b^ All enrolled except those transferred out of clinic before the end of time period are included in denominator.

Among those who were on ART for less than three years at study start (9.9% of the sample), ITT 24‐month retention in care was substantively lower in the intervention arm (risk difference: 9.6, 95% CI: −17.5 to −1.8%, Table S3). However, the intervention arm retained higher ITT VL completion and suppression (Table S4).

Primary outcomes remained similar when stratifying by facility‐based or community‐based ACs, or by whether or not participants had documented VL suppression in the 18 months before study start (Tables S3 and S4).

During the first year of the study, SoC AC patients were more likely to have a VL result available for their clinical consultation AC visit (92.4% vs. 88.3%). During the second year of the study, intervention arm patients were more likely to have VL results available for their clinical visit (86.4% vs. 81.8%) (Table S5).

## 4 Discussion

In this cluster‐randomized trial of extended ART dispensing intervals, AC patients receiving six‐month ART refills showed non‐inferior 24‐month retention in care, VL completion and VL suppression to those in SoC ACs.

This is the first study reporting 24‐month outcomes. Our outcomes are consistent with interim 12‐month outcomes in two recently published cluster randomized trials in Zimbabwe [31] and Lesotho [30] that showed non‐inferior retention in community‐based DSDs receiving six‐monthly refills, compared to three‐monthly facility controls. VL suppression was non‐inferior in the Lesotho study [30], but not the Zimbabwean study, possibly because of low VL completion rates [31].

In this study, retention in care and viral suppression were, unsurprisingly, high overall with both arms achieving greater than 92% 24‐month retention and viral suppression among those with complete VLs. Patients were treatment experienced, with a median time on ART of over seven years. In addition, all of the patients were required to be clinically stable to enrol in ACs. Patients’ continued retention and viral suppression were expected but it is reassuring that extending their refill length and reducing the frequency of their group AC meetings, thereby reducing interactions with the health system and their peers, did not impact their 24‐month outcomes.

Retention at 24 months was similar to community adherence clubs in Kwa‐Zulu Natal reporting 94.9% 12 to 24 month retention [41, 42]. Twelve‐month retention (>97%) was similar to previous Western Cape AC studies that reported 95% to 97% [8, 35, 43, 44], and higher than 89.5% reported from a South African AC model evaluation comparing 24 randomly allocated intervention and control facilities undertaken in Gauteng, Kwa‐Zulu Natal, Limpopo and North West provinces at the same time point [9]. Retention was also comparable to the six‐monthly dispensing arms of the recently published non‐inferiority trials from Zimbabwe (93.6%) and Lesotho (94.7%) [30, 31].

VL completion was high in the first 12 months, and lower in the second year of the study (85.2% in SoC and 90.8% in intervention), but still equivalent or better than published data on similar AC cohorts (85.2% [43], 79.7% to 86.5% [7], 82% to 85% [14]).

Viral suppression for those with VLs in the first and second years of the study was also similar to previous Western Cape AC studies (94% to 97%) [8, 43, 44, 45] and the aforementioned South African evaluation (95.2%) [9]. For the purposes of this study, the intervention ACs had a specific, individual VL visit at the facility separate from their AC visits. In developing the study protocol, this visit was considered critical to ensure that a recent VL result was available at the annual clinical review, similar to SoC AC patients. With such high viral suppression, the additional burden placed on the intervention AC patients to attend the clinic for their blood draw before their clinical consultation may not be warranted and alternatives should be considered for programmatic implementation. For example, VLs could be taken at the previous AC visit. However, until VLs can be taken at community venues through point of care or dry blood spot technologies, this would require all AC visits to take place at the facility. Alternatively, VLs could be taken at the annual clinical consultation AC visit, which would avoid the need for point of care technology or for shifting all ACs to be facility‐based but would mean a suboptimal clinical visit without a VL result. In both scenarios it would be important to stress a reliable recall system for the few AC patients who experience viral rebound.

Our results add to the growing literature showing good retention and clinical outcomes associated with extended ART dispensing intervals [30, 31, 46, 47, 48]. This is particularly important at a time when countries will be considering whether to continue or reverse six‐monthly ART refills provided during the COVID‐19 pandemic. We had high rates of enrolment, no crossover between groups, and results consistent across community and facility ACs. The study was done within routine ACs at a large public ART clinic and its catchment area, providing strong real‐world evidence that extending ART dispensing intervals is safe if patients want access to six‐month ART refills. These findings may be generalizable to group DSD models where patients have been on ART and retained within their DSD model for a number of years.

Reducing the frequency of AC visits ostensibly reduces the opportunity for peer and facilitator support, which patients have described as an important component of the AC model [10, 13]. Paradoxically, in a qualitative sub‐study of our trial, patients reported that less frequent visits increased peer and facilitator support as AC members were able to set aside time for the visit and peer interaction if it was only twice a year [49]. Our sub‐analysis of AC patients on ART for less than three years at baseline suggests that extended dispensing intervals may result in inferior retention for this group, indicating more frequent group contact initially may support less frequent interaction later on. However, our sample was too small to draw any conclusions. Future research should explore extended dispensing intervals in newly stable patients, and whether these results hold in subpopulations not well represented by this sample, such as youth, and other contexts, such as rural settings.

South Africa’s DSD policy to date has been conservative towards empowering stable ART patients with a choice to extend their refills. At a time when most sub‐Saharan African countries had moved to a minimum of three‐monthly ART refills, South Africa continued to limit refill length to two months. In 2020, the updated DSD policy shifted to three‐monthly but, in practice, remained constrained by the Central Chronic Medication Dispensing and Distribution (CCMDD), the main DSD drug supply system, continuing to only enable two‐monthly refills. This policy remained unchanged in South Africa’s guidance during COVID‐19 [50]. Now with this evidence from a South African trial, the world’s biggest ART programme should rapidly make longer ART refills available within their DSD program to continue supporting long‐term adherence critical to both the health of people living with HIV and the prevention benefit to those not yet infected. While the further research proposed remains necessary, there is sufficient evidence and global guidance for six‐monthly refills to become policy in South Africa.

This study had a number of limitations. First, we included some participants without documentation of recent viral suppression. However, the sensitivity analyses suggest this did not impact on the results. Second, AC patients who missed a scheduled visit by more than five days were not always referred back to mainstream care at their clinic but were allowed to continue their care within their AC. For purposes of the analysis, we treated these patients as not retained in AC care, but their continued care within the AC model may have supported their overall retention. As there was a higher proportion of these patients in SoC ACs, this may have benefitted retention for the SoC arm, but may have reduced SoC viral suppression. Third, during the first year of the study a once‐off non‐routine four‐month ART refill period was provided to all SoC AC patients in anticipation of a potential Cape Town water crisis [51, 52], in addition to the routine four‐month refill at year end. This made the SoC slightly atypical, but constituted only one fewer visit in this arm over the 24‐month period. Fourth, we were well powered to detect 3.1% difference in retention in care, but only a 6.5% and 6.7% reduction in VL completion and suppression respectively. However, given the substantively higher VL completion and suppression (RD: 5.5%, 95% CI: 1.5 to 9.5 and 4.6%; 95% CI: 0.2 to 9 respectively) in the intervention arm, the direction of this effect is unlikely to change with increased study power. Lastly, the intervention AC had more involvement by the study team. While AC procedures were standardized, and the same facilitators ran both SoC and intervention ACs and were the primary point of contact for patients across both arms, a nurse employed by the study team was more involved with the clinical procedures for the intervention arm, introducing the potential for a higher standard of clinical care, including VL completion.

## 5 Conclusions

This study demonstrated that patients receiving their care through DSD models, who want less frequent visits and longer multi‐month dispensing, can be safely transitioned to six‐monthly visits and ART refills without compromising their longer term retention and viral suppression outcomes.

## Competing Interests

The authors have no competing interests to declare.

## Authors’ Contributions

LW and AG conceptualized the study. TC and KL managed and curated data and TC performed the analysis. LW and RG were responsible for funding acquisition. TC, LW, AG, CMK and CO contributed to methodology. CMK, TM, KL and NZ ran the study. TC and LW drafted the initial manuscript. All authors reviewed and approved the final manuscript.

## Abbreviations

AC, adherence club; ART, anti‐retroviral therapy; DSD, differentiated service delivery; GEE, generalized estimating equations; ICC, intra‐cluster correlation; IQR, interquartile range; MSF, Médecins Sans Frontières; PEPFAR, U.S. President's Emergency Plan for AIDS Relief; RD, risk difference; SoC, standard of care; SOP, standard operation procedure; VL, viral load; WCDoH, Western Cape Provincial Department of Health.

## Supporting information


**Figure S1.** Intention to treatrist differences (95% CI).

**Table S1.** Study power estimates for a range of intervention effects and intra‐cluster correlation using actual sample size, cluster size and retention in care estimates of the SoC arm
**Table S2.** Reasons for not being retained in AC care
**Table S3.** Sensitivity analyses of retention at 12 and 24 months
**Table S4.** Sensitivity analyses of viral load completion suppression
**Table S5.** Timing of blood draws for routine viral load monitoring
