# Supplementary figures and images for: Twenty‐four‐month outcomes from a cluster‐randomized controlled trial of extending antiretroviral therapy refills in ART adherence clubs
Source: J Int AIDS Soc. 2020 Dec 19;23(12):e25649. doi: 10.1002/jia2.25649 (PMC7749539; doi:10.1002/jia2.25649)

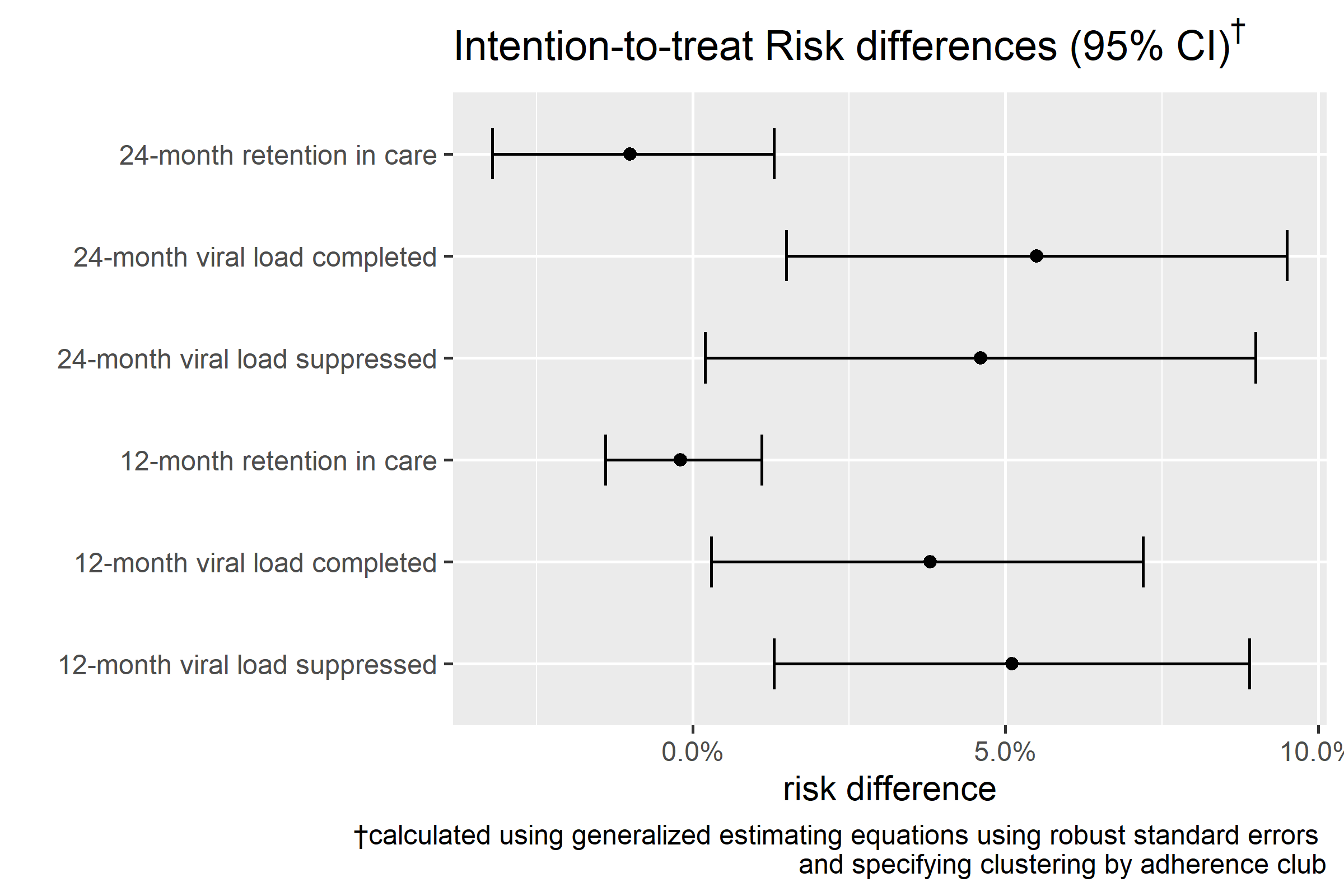

Supplement: Supplementary file 1 — Figure S1. Intention to treatrist differences (95% CI). [file JIA2-23-e25649-s001.tiff]
