# Supplementary material for: Twenty‐four‐month outcomes from a cluster‐randomized controlled trial of extending antiretroviral therapy refills in ART adherence clubs
Source: J Int AIDS Soc. 2020 Dec 19;23(12):e25649. doi: 10.1002/jia2.25649 (PMC7749539; doi:10.1002/jia2.25649)
Supplement: Supplementary file 2 — Table S1. Study power estimates for a range of intervention effects and intra‐cluster correlation using actual sample size, cluster size and retention in care estimates of the SoC arm Table S2. Reasons for not being retained in AC care Table S3. Sensitivity analyses of retention at 12 and 24 months Table S4. Sensitivity analyses of viral load completion suppression Table S5. Timing of blood draws for routine viral load monitoring [file JIA2-23-e25649-s002.docx]

Supplementary table 1. Study power estimates for a range of intervention effects and intra-cluster correlation using actual sample size, cluster size and retention in care estimates of the SoC arm

| Outcome  (24 months) | Average cluster size in the intervention | Average cluster size in the SoC | Observed proportion in SoC | ICC | **Study power^†^**  Effect size (Risk Difference): | | | | | | |
| --- | --- | --- | --- | --- | --- | --- | --- | --- | --- | --- | --- |
|  |  |  |  |  | 1% | 2% | 3% | 4% | 5% | 6% | 7% |
| Retention | 24.4 | 24.4 | 93.6% | 0.004917 | 21.9% | 51.4% | 78.7% | 93.5% | 98.6% | 99.8% | 100.0% |
| VL completion | 24.4 | 24.4 | 85.2% | 0.061841 | 10.9% | 20.3% | 33.0% | 47.6% | 62.2% | 75.0% | 84.9% |
| VL Suppression | 24.4 | 24.4 | 82.6% | 0.058055 | 10.6% | 19.4% | 31.3% | 45.3% | 59.6% | 72.6% | 82.9% |
| Abbreviations: ICC (intracluster correlation), SoC (standard of care), VL (viral load)  ^†^Calculated using Stata 15 *power* function for onesided test of two proportions, alpha=0.05 | | | | | | | | | | | |

Supplementary table 2. Reasons for not being retained in AC care

|  | **SoC**  **N=421**  **N (%)** | **Intervention**  **N=244**  **N (%)** |
| --- | --- | --- |
| Missed scheduled AC visit by more than 5 days but returned to facility within 90 days | 295 (70.1%) | 142 (58.2%) |
| *Of whom returned to AC^†^* | *172 (40.9%)* | *49 (20.1%)* |
| High viral load | 26 (6.2%) | 23 (9.4%) |
| Transferred out of facility | 22 (5.2%) | 15 (6.1%) |
| Pregnancy | 21 (5.0%) | 21 (8.6%) |
| Missed scheduled AC visit by more than 90 days, lost from clinic care | 22 (5.2%) | 22 (9.0%) |
| Tuberculosis | 5 (1.2%) | 2 (0.8%) |
| Died | 2 (0.5%) | 2 (0.8%) |
| Other/unknown | 23 (5.5%) | 13 (5.3%) |

Abbreviations: AC (Adherence Club), SoC (standard of care)

^†^ for the purposes of this study, defined as not retained in AC care

Supplementary table 3. Sensitivity analyses of retention at 12 and 24 months

|  |  | **SoC clubs**  **n/N (%)** | **Intervention clubs**  **n/N (%)** | **ICC** | **Risk difference (95% CI)**^†^ **Intervention vs. SoC** |
| --- | --- | --- | --- | --- | --- |
| Main model (ITT) | 12-month retention | 1146/1173 (97.7%) | 953/977 (97.5%) | <0.001 | -0.2% (-1.4 to 1.1) |
|  | 24-month retention | 1098/1173 (93.6%) | 905/977 (92.6%) | 0.005 | -1.0% (-3.2 to 1.3) |
| Excluding no documented VL suppression at baseline | 12-month retention | 1095/1120 (97.8%) | 905/925 (97.8%) | <0.001 | 0.1% (-1.2 to 1.4) |
|  | 24-month retention | 1054/1120 (94.1%) | 859/925 (92.9%) | 0.007 | -1.2% (-3.5 to 1.0) |
| Including only no documented VL suppression at baseline | 12-month retention | 51/53  (96.2%) | 48/52 (92.3%) | <0.001 | -3.9% (-12.3 to 4.6) |
|  | 24-month retention | 44/53  (83.0%) | 46/52 (88.5%) | 0.020 | 5.4% (-6.9 to 17.8) |
| Only those <3 years on ART at baseline | 12-month retention | 115/120 (95.8%) | 86/93 (92.5%) | <0.001 | -4.0% (-10.2 to 2.2) |
|  | 24-month retention | 111/120 (92.5%) | 79/93 (84.9%) | <0.001 | -9.6% (-17.5 to -1.8) |
| Facility ACs | 12-month retention | 591/606 (97.5%) | 529/543 (97.4%) | 0.006 | -0.1% (-2.1 to 1.9) |
|  | 24-month retention | 569/606 (93.9%) | 506/543 (93.2%) | 0.013 | -0.7% (-3.9 to 2.5) |
| Community ACs | 12-month retention | 555/567 (97.9%) | 424/434 (97.7%) | <0.001 | -0.2% (-1.7 to 1.4) |
|  | 24-month retention | 529/567 (93.3%) | 399/434 (91.9%) | <0.001 | -1.4% (-4.5 to 1.8) |
| Abbreviations: AC (Adherence Club), ICC (intra-cluster correlation), ITT (intention-to-treat), SoC (standard of care)  ^†^Calculated using generalized estimating equations using robust standard errors and specifying clustering by adherence club | | | | | |

Supplementary table 4. Sensitivity analyses of viral load completion suppression

|  |  | **Intention to treat^†^** | | | | | **Modified intention to treat^‡^** | | | | **Completed viral load** | | | |
| --- | --- | --- | --- | --- | --- | --- | --- | --- | --- | --- | --- | --- | --- | --- |
|  |  | **SoC** | | **intervention** | **ICC** | **RD^§^**  **(95% CI)** | **SoC** | **intervention** | **ICC** | **RD^§^**  **(95% CI)** | **SoC** | **intervention** | **ICC** | **RD^§^**  **(95% CI)** |
| 12 months  (primary analysis) | N | 1173 | | 977 | - | **-** | 1167 | 975 |  |  | 1107 | 956 | - | **-** |
|  | VL completed | | 1107 (94.4%) | 956 (97.9%) | 0.166 | 3.5%  (-0.1 to 7.1) | 1106 (94.8%) | 955 (97.9%) | 0.178 | 3.2%  (-0.3 to 6.6) | - | - | - | - |
|  | VL suppressed | | 1065 (90.8%) | 933 (95.5%) | 0.111 | 4.7%  (0.7 to 8.7) | 1064 (91.2%) | 932 (95.6%) | 0.115 | 4.4%  (0.5 to 8.4) | 1065 (96.2%) | 933 (97.6%) | 0.019 | 1.4%  (-1.9 to 4.7) |
| 24 months (primary analysis) | N | | 1173 | 977 | - | - | 1160 | 963 |  |  | 999 | 887 | - | - |
|  | VL completed | | 999 (85.2%) | 887 (90.8%) | 0.062 | 5.6%  (1.3 to 9.9) | 996 (85.9%) | 879 (91.3%) | 0.061 | 5.4%  (1.2 to 9.6) | - | - | - | - |
|  | VL suppressed | | 969 (82.6%) | 853 (87.3%) | 0.058 | 4.7%  (0 to 9.4) | 967 (83.4%) | 847 (88.0%) | 0.059 | 4.6%  (0.0 to 9.2) | 969 (97.0%) | 853 (96.2%) | 0.008 | -0.8%  (-3.3 to 1.7) |
| 24 months only those <3 years on ART at baseline | N | | 120 | 93 | - | - | 118 | 91 | - | - | 97 | 79 | - | - |
|  | VL completed | | 97 (80.8%) | 79 (84.9%) | <0.001 | 4.1%  (-6 to 14.2) | 96 (81.4%) | 78 (85.7%) |  | 4.4%  (-5.7 to 14.4) | - | - | - | - |
|  | VL suppressed | | 93 (77.5%) | 77 (82.8%) | 0.021 | 5.3%  (-6.2 to 16.8) | 93 (78.8%) | 77 (84.6%) | 0.054 | 5.8%  (-5.5 to 17.1) | 93 (95.9%) | 77 (97.5%) | 0.581 | 1.6%  (-3.7 to 6.9) |
| 24 months excluding no documented VL suppression at baseline | N | | 1120 | 925 | - | - | 1110 | 911 |  |  | 958 | 846 | - | - |
|  | VL completed | | 958 (85.5%) | 846 (91.5%) | 0.061 | 5.9%  (1.7 to 10.1) | 957 (86.2%) | 838 (92.0%) | 0.063 | 5.8%  (1.6 to 9.9) | - | - | - | - |
|  | VL suppressed | | 934 (83.4%) | 816 (88.2%) | 0.060 | 4.8%  (0.2 to 9.5) | 933 (84.1%) | 810 (88.9%) | 0.064 | 4.9%  (0.3 to 9.4) | 934 (97.5%) | 816 (96.5%) | 0.011 | -1.0%  (-3.4 to 1.3) |
| 24 months including only no documented VL suppression at baseline | N | | 53 | 52 |  |  | 50 | 52 |  |  | 41 | 41 |  |  |
|  | VL completed | | 41 (77.4%) | 41 (78.8%) | 0.169 | 1.2% (-16.7 to 19.0) | 39 (78.0%) | 41 (78.8%) | 0.129 | 0.7% (-16.5 to 18.0) |  |  |  |  |
|  | VL suppressed | | 35 (66.0%) | 37 (71.2%) | 0.060 | 5.6% (-13.0 to 24.2) | 34 (68.0%) | 37 (71.2%) | 0.007 | 3.2% (-14.9 to 21.3) | 35 (85.4%) | 37 (90.2%) | <0.001 | 3.2% (-10.1 to 16.5) |
| 24 months facility ACs | N | | 606 | 543 | - | - | 603 | 539 | - | - | 517 | 500 | - | - |
|  | VL completed | | 517 (85.3%) | 500 (92.1%) | 0.115 | 6.8%  (1.0 to 12.5) | 517 (85.7%) | 497 (92.2%) | 0.107 | 6.5%  (0.8 to 12.2) | - | - | - | - |
|  | VL suppressed | | 503 (83.0%) | 481 (88.6%) | 0.094 | 5.6%  (-0.4 to 11.6) | 503 (83.4%) | 478 (88.7%) | 0.087 | 5.3%  (-0.7 to 11.2) | 503 (97.3%) | 481 (96.2%) | <0.001 | -1.1%  (-5.2 to 3.0) |
| 24 months community ACs | N | | 567 | 434 | - | - | 557 | 424 | - | - | 482 | 387 |  |  |
|  | VL completed | | 482 (85.0%) | 387 (89.2%) | 0.008 | 4.2%  (-0.2 to 8.5) | 479 (86.0%) | 382 (90.1%) | 0.012 | 4.1%  (-0.1 to 8.3) | - | - | - | - |
|  | VL suppressed | | 466 (82.2%) | 372 (85.7%) | 0.021 | 3.5%  (-1.6 to 8.6) | 464 (83.3%) | 369 (87.0%) | 0.029 | 3.7%  (-1.2 to 8.7) | 466 (96.7%) | 372 (96.1%) | 0.017 | -0.6%  (-3.3 to 2.1) |

Abbreviations: AC (Adherence Club), ICC (intra-cluster correlation), SoC (standard of care), RD (risk difference), VL (viral load)

† All enrolled are included in the denominators; ‡ all enrolled except those transferred out of clinic before end of time period are included in denominator;

**^§^**Risk differences calculated using generalized estimating equations using robust standard errors and specifying clustering by adherence club

Supplementary table 5. Timing of blood draws for routine viral load monitoring

|  | SoC  N=1,173  n (%) | | Intervention  N=977 n (%) |
| --- | --- | --- | --- |
| 12-month viral loads (completed 0-12 months after first visit) | | | |
| After study start, before clinical consultation AC visit | 1084 (92.4%) | | 863 (88.3%) |
| On scheduled blood draw date | 913 (77.8%) | | 605 (61.9%) |
| Median time between blood draw and clinical consultation visit, days (IQR) | 95.5^†^ (56-112) | | 14 (14-14) |
| At clinical consultation AC visit (results not available for clinical consult, 2 days before to 7 days after clinical consultation) | | 15 (1.3%) | 72 (7.4%) |
| After clinical visit, but within first 12 months | | 8 (0.7%) | 21 (2.1%) |
| Viral load not done | | 66 (5.6%) | 21 (2.1%) |
| 24-month viral loads (completed 12-24 months after first visit) | | | |
| After study start, before clinical consultation AC visit | | 960 (81.8%) | 844 (86.4%) |
| On scheduled blood draw date | | 780 (66.5%) | 686 (70.2%) |
| Median time between blood draw and clinical consultation visit, days (IQR) | | 56 (56-56) | 50^‡^ (32-54) |
| At clinical consultation AC visit (results not available for clinical consult, 2 days before to 7 days after clinical consultation) | | 30 (2.6%) | 10 (1.0%) |
| After clinical visit, but within first 12 months | | 9 (0.8%) | 33 (3.4%) |
| Viral load not done | | 174 (14.8%) | 90 (9.2%) |

Abbreviations: Adherence Club (AC), IQR (interquartile range), SoC (standard of care)
^†^longer time between blood draw and clinical visit as a result of 4-month ART refill during water crisis

^‡^blood draws were deliberately spaced out so as not to coincide with other ACs’ clinical visits


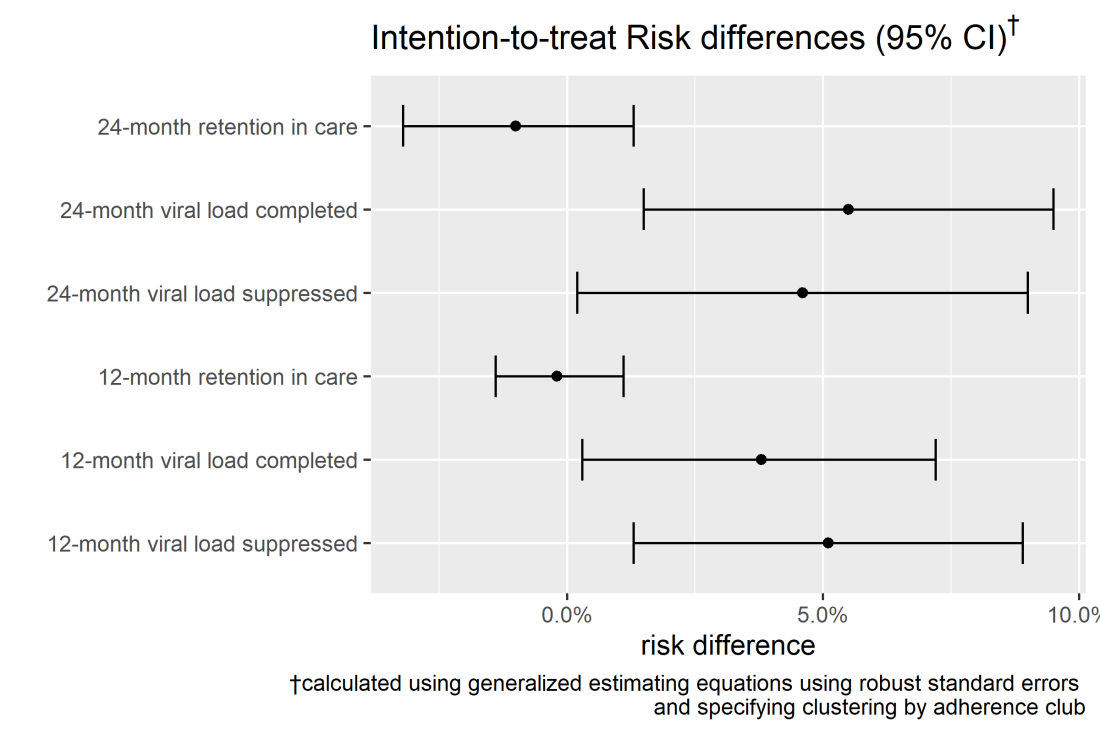


Supplementary figure 1. 12- and 24-month retention in care, viral load completion, and viral load suppression risk differences
